# Will the new decade bring home remote monitoring of heart failure patients?

**DOI:** 10.1007/s12471-019-01359-z

**Published:** 2019-12-16

**Authors:** Y. M. Pinto

**Affiliations:** Department of Cardiology, Academic Medical Center, University of Amsterdam, Amsterdam, The Netherlands

This issue of the Netherlands Heart Journal contains two articles on management of heart failure [1, 2]. The guideline on heart failure treatment suggests that management of heart failure is fairly straightforward: aiming for maximal medical therapy and at certain disease severity device therapy may be added, all of which can be summarised in a single algorithm to fit all patients with reduced ejection fraction [3, 4]. It seems therefore paradoxical that there is a need to monitor heart failure patients almost continuously and adjust therapy according to objective measures. The reason seems to be that heart failure can deteriorate unexpectedly and spiral down towards life-threatening pump failure or, possibly, lethal arrhythmias despite appropriate medication and adherence to the guidelines. As a result, the clinicians who treat and follow these patients search for objective measures that allow them to see these derailments coming and, most importantly, avert them, thereby avoiding the dreaded outcomes of heart failure.

One of the recently developed tools for monitoring patients, the CardioMEMS, is an implantable sensor that continuously monitors the pressure in the pulmonary artery as an indicator of cardiac filling pressures.

However, the benefit of advanced diagnostics such as continuous pressure monitoring in the pulmonary artery relies on the ability to avert the worsening it predicts. In recent years, we have seen an increased number of options to intervene pharmacologically, such as the recently added combination of angiotensin receptor blockade with neprilysin inhibition [5]. Last years the number of ways to treat HF patients increased with added medical and device options. One may anticipate that this calls for objective measures to monitor the effect of these added measures or even to choose among them. However, currently there is no evidence supporting the use of diagnostic measures, such as CardioMEMS, to select between these therapies. So the selection is based on the clinical characteristics of the patients who were included in the trials where they were shown to be efficacious.

Veenis and Brugts summarise the evidence for remote monitoring of heart failure patients, which shows the challenges to demonstrate significant gain [1]. This might suggest that even when detection of imminent deterioration is achieved, the subsequent clinical intervention in response to these signs needs to differ enough from already applied measures to have a clear clinical impact.

In this same issue, Brugts and colleagues describe a national prospective study, the MONITOR HF trial, to assess the potential benefit of continuous remote monitoring of pressure in the pulmonary artery in heart failure patients [2]. This has been shown earlier to decrease hospitalisations for worsening heart failure [6]. The CHAMPION trial enrolled subjects with NYHA functional class III heart failure, irrespective of left ventricular ejection fraction, which is very similar to the type of patients the MONITOR HF trial will enrol.

To obtain data from a similar study in the Netherlands is of great value. First of all, in the CHAMPION trial the main benefit at 15 months was decreased admissions for worsening heart failure without a mortality benefit. Roughly speaking, CardioMEMS decreased the number of admissions by one or two admissions per patient per year. Therefore, a relevant question is whether—apart from the obvious patient benefit—the costs of the device and its continuous monitoring are offset by saving admissions. This is not obvious since costs of admission differ considerably across countries, so the cost-benefit equation of this effect may not be similar across all countries.

In addition, the ability to predict imminent decompensation and how to intervene also differs across countries. Therefore, the added role of remote monitoring may differ between, for example, the US and the Netherlands. In the Netherlands, the existing heart failure outpatient clinics form a tightly woven fabric that provides high-quality care to guide and monitor each heart failure patient. This may decrease the ability of added remote monitoring to further improve upon this. In countries where this type of care cannot be as tightly organised, for instance due to large distances and a different health-care system, the added benefits of remote monitoring may differ significantly.

Therefore, it will be of great interest to see how the continuous measurement of pulmonary artery pressures is translated into clinical decisions in the Dutch health-care system and whether this will lead to similar benefits as experienced elsewhere. Finally, whether it will be successful will, to a considerable extent, depend on how doctors respond to increasing filling pressures and whether their immediate interventions (e.g. increase diuretics) actually translate into longer term beneficial effects.
